# Penetrating carotid artery injury by air rifle: a case report

**DOI:** 10.1186/s13256-023-04080-z

**Published:** 2023-08-14

**Authors:** Summer Hassan, Sergei Tchijov

**Affiliations:** 1 Department of Vascular Surgery, Middlemore Hospital, Auckland University, 100 Hospital Road, Auckland, New Zealand; 2 University of Auckland, Auckland, New Zealand

**Keywords:** Carotid, Penetrating vascular injury, Carotid trauma, Vascular repair

## Abstract

**Background:**

Air rifle injuries can cause significant vascular injuries. This air rifle injury has resulted in a penetrating neck trauma traversing the common carotid artery. There is debate around the need for radiological investigation, the most appropriate investigational modality, and the need for surgical exploration versus a conservative approach. This case report aims to exemplify a successful approach to managing Penetrating Carotid Injuries (PCI) while shedding light on the rationale behind the management decisions.

**Presentation:**

An 18-year-old Caucasian man arrived at the hospital following an air rifle injury to the right side of the neck, with active bleeding and a moderate haematoma displacing the trachea. He was haemodynamically stable, with a Glasgow Coma Scale (GCS) of 15 and no evidence of bruit. Computed Tomography Angiography (CTA) showed Right common carotid (CCA) artery injury with associated post-traumatic pseudoaneurysm. The pellet trajectory traverses the right superior thyroid gland. A duplex ultrasound scan (USS) confirmed two areas of arterial blush at the right CCA. Management involved neck exploration under General Anaesthesia (G.A.), repair of right CCA, bullet extraction, and wound washout. He received antibiotics for ten days and a single agent of antiplatelets for three months and was discharged two days postoperatively with no complications. He was followed up for eight months with no evidence of any trauma sequelae.

**Conclusion:**

Penetrating carotid artery injuries are a serious concern. The small-sized pellets carry the risk of embolization. Therefore, neck exploration remains the gold standard treatment for PCI. Appropriate operative planning is crucial and can be optimised using radiological diagnostic modalities in haemodynamically stable patients. CTA is a non-invasive, swift, and adequate alternative to arteriography, providing valuable diagnostic information on vascular and aerodigestive injuries and bullet trajectory. This enables appropriate preparedness to achieve excellent outcomes in such critical cases.

## Background

The damage inflicted by air rifles is often underestimated. In this case, air rifle injury has resulted in a penetrating neck trauma traversing the common carotid artery. There is debate around the need for radiological investigation, the most appropriate investigational modality, and the need for surgical exploration versus a conservative approach. This case report aims to exemplify a successful strategy in managing Penetrating Carotid Injuries (PCI) while shedding light on the rationale behind the management decisions.

## Case presentation

An 18-year-old Caucasian man arrived by ambulance at Middlemore Hospital after an air rifle injury to his neck. The entrance wound was to the right of the midline anteriorly. He was haemodynamically stable and had a Glasgow Coma Scale (GCS) of 15. The entry wound was located right to the midline of the neck; no exit wounds were noted. There was a small amount of active bleeding, subcutaneous emphysema, and a moderate haematoma displacing the trachea to the left. The examination was otherwise unremarkable, with no detectable bruit. The patient’s blood tests were unremarkable. He proceeded for CTA of the carotid arteries following 80 ml Omnipaque 350 IV. This showed a metal pellet approximately 6.57 mm long, lodged 1.2 cm behind the mid-right common carotid artery (CCA) and about 3.5 cm proximal to the carotid bifurcation in front of the transverse process of the C6 vertebra. The pellet tract appeared to have passed through the superior right thyroid lobe. There were features suggestive of post-traumatic pseudoaneurysms of the right CCA.

Additionally, right neck soft tissue swelling and haematoma with a leftward displacement of the trachea and lower pharynx were evident. Primary resuscitation was initiated in the emergency department. The patient proceeded to the theatre for urgent neck exploration, repair of the CCA, retrieval of the pellet, and washout of the wounds. He was positioned supine under general anaesthesia. The top end of the table was elevated 20 degrees, and the patient’s head was turned to the left with the neck extended. Two grams of Cefazolin were administered on induction. The right carotid artery was interrogated with a duplex scan which demonstrated two areas of blush anteriorly and posteriorly, confirming the presence of the pseudoaneurysm.

The patency of common, internal, and external carotid arteries was confirmed. The location of the carotid bifurcation and the arterial injury was marked on the skin. The right Great Saphenous Vein (GSV) was also scanned and marked proximally in case a graft is required. The neck and groin were prepared and draped, followed by a 12 cm longitudinal incision along the anterior border of the sternocleidomastoid muscle. The dissection was complicated by significant tissue plane distortion by the present haematoma. The proximal CCA was exposed above the anterior belly of the omohyoid muscle and encircled with a vessel loop, and the proximal internal and external carotid arteries were exposed and encircled with vessel loops. After the arteries were ready to be clamped, the patient was given 5000 IU of Heparin intravenously. After three minutes of systemic circulation, we explored the CCA distal to the pseudoaneurysm. The artery was dissected and encircled with a vessel loop. The Vagus nerve was visualised and protected throughout the procedure. Afterward, CCA was clamped distally and proximally with DeBakey clamps.

**Fig. 1:**
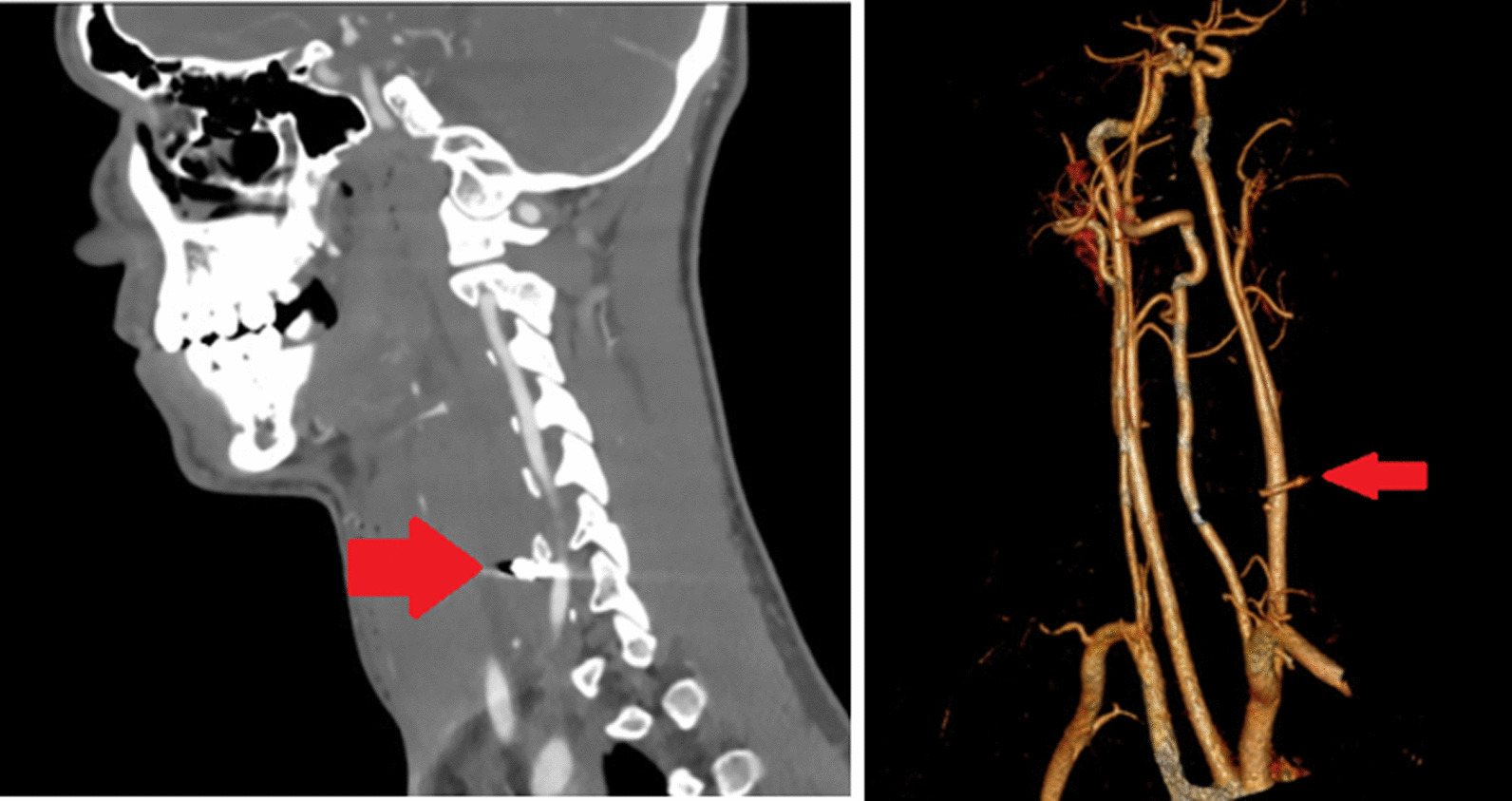
CT images and 3D reconstruction showing the projectile going through the anterior medial wall and exiting through the posterior wall of the carotid artery. Arrow demonstrating the pellet

**Fig. 2:**
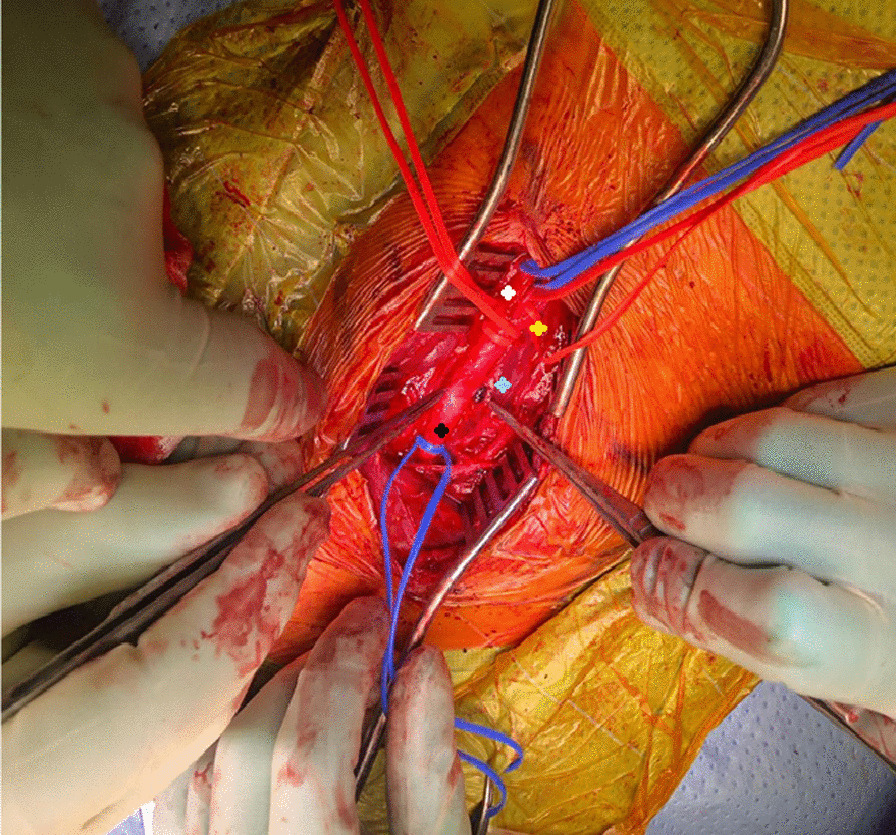
Intraoperative image showing: Black: Right Common Carotid Artery. White: Right External Carotid Artery. Yellow: Right Internal carotid artery. Blue: Pellet

**Fig. 3:**
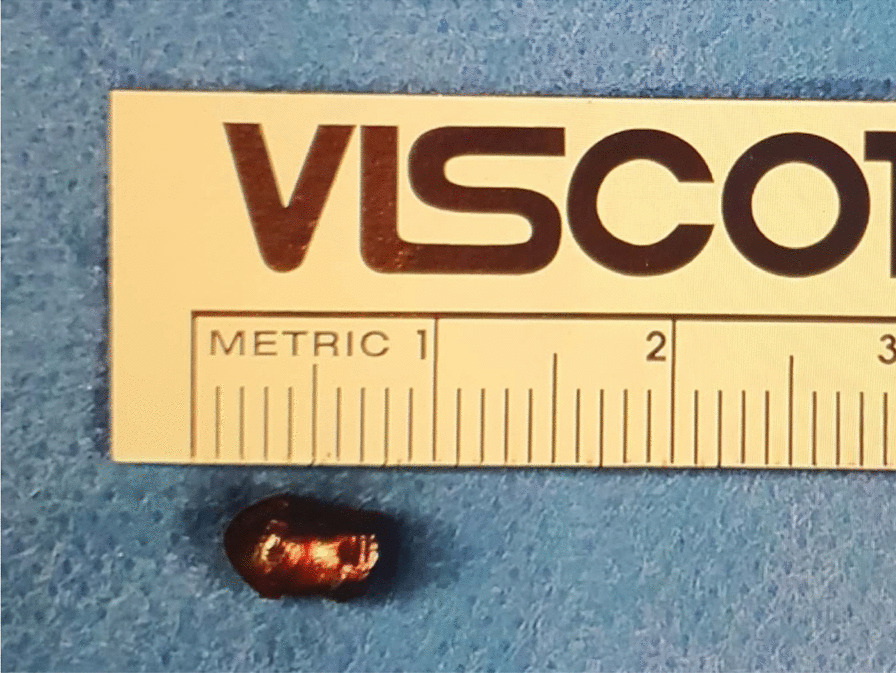
The explanted pellet

After three minutes of observing the cerebral oximetry parameters, no changes from baseline were noted. The procedure was therefore continued without shunting. The injury site was accessed through the overlying haematoma. The projectile appeared to go in via the anterior medial wall. It exited through the posterior wall of the carotid artery, with both entry and exit wounds occluded by clots and no active bleeding (Fig. 1). The clots were removed. Both wounds were approximately 3 mm in size with healthy-looking arterial edges. The lumen of the artery was satisfactorily visualised through both wounds; there were no clots or foreign bodies found inside. The inflow and backflow were strong and pulsatile. The artery was generously flushed with heparinised saline, and the wounds were repaired primarily with continuous 6/0 Prolene sutures in a transverse direction without any tension. The repairs were flushed and vented before completion, and the artery was allowed into circulation using the standard sequential unclamping technique. Two 6/0 Prolene rescue sutures were required to achieve haemostasis. The lead pellet was found lodged deeper behind the carotid artery in front of the transverse process of the 6th vertebra. It was taken out (Figs. 2, 3). An injury to the aerodigestive tracts seemed very unlikely, considering the trajectory of the pellet and the intraoperative findings. After discussing the operative results with the general surgical team, no further exploration was conducted.

The neck haematoma was washed out with Normal Saline. The small skin entry wound was incised in a transverse direction, explored, washed out with normal saline, and closed. Then, a 10 Fr Redivac drain was inserted. The wound was infiltrated with 0.5% Marcain with adrenaline. During the surgery, the blood loss was minimal. The patient woke up in the operating room with no neurological deficits. He was to the High Dependency Unit to the surgical ward the next day. He was started on Aspirin 100 mg orally and continued taking it for three months. He also completed a 10-day course of Augmentin. The drain was removed on the second postoperative day. The patient was discharged on the same day with recommendations to maintain light work duties and to visit his family doctor in 10 days for a wound review. He was contacted six weeks following his surgery and reported feeling well and ready to resume full duties at his work. He had a duplex USS of his right carotid vessels showing a mild degree of residual extraluminal circumferential hypoechoic thickening at the mid-common carotid artery to the carotid bifurcation. He had normal thyroid function and did not attend a scheduled thyroid USS organised in the outpatient settings. He was reviewed again three months postoperatively and was symptom-free. His antiplatelet medication was stopped at this point, and a repeat duplex revealed complete resolution of the previously noted extraluminal thickening and good triphasic flows throughout the carotid artery. The last formal contact was eight months postoperatively with a repeat negative duplex. He was therefore discharged with no further follow-up.

## Discussion and conclusions

Penetrating neck injuries form 10% of all penetrating traumas, with a concomitant carotid artery injury in up to 20% of these cases [1]. Penetrating carotid injuries (PCI) are caused mainly by stab wounds and ballistic injuries, followed by self-harm and motor vehicle collisions [2, 3]. These injuries can lead to mortality rates reaching 100% if left untreated [4]. One of the aims of this presentation is to raise the public and the surgical community's awareness of the danger airguns represent. Apart from other serious sequelae, vascular injuries caused by small-sized airgun pellets carry the risk of embolisation and the resulting downstream organ ischemia [5–7]. Despite comparatively strict regulations against firearms in New Zealand, air rifle regulations are much looser. They allow anyone under 18 years old to use an airgun under the “immediate supervision” of a person aged 18 or older [8].

Similarly, in the U.K., the use of airguns is permitted from the age of 14 without supervision and with supervision under the age of 14 [9]. Detailed interrogation of the multitude of vital neck structures by physical examination is limited, and serious injuries can be missed [10, 11]. There is debate on the best management approach in haemodynamically stable patients with penetrating neck injuries. Some surgeons opt to proceed with neck exploration when injuries violate the platysma muscle and believe this approach to be safe with low complication rates [11, 12]. These mandatory explorations, however, were challenged in the 1980s due to the high rate of nontherapeutic surgeries, the increased hospital length of stay, and the emerging alternative approach of selective conservative management [13–15]. Despite the variability in management plans for penetrating neck injuries, there is a consensus that a penetrating carotid injury, as in this case, requires urgent surgical exploration [4]. Surgical intervention to repair PCI requires tactical planning to protect the brain from ischemic injuries should the need for ICA clamping arise. An assessment of cerebral perfusion by measuring backflow pressure in the distal ICA, a visual estimation of the quality of ICA back bleeding, or using cerebral oximetry is currently helping make decisions regarding selective carotid shunting.

Routine shunting is also advocated for by many. Cerebral oximetry monitoring was utilised in the presented case. Furthermore, the foreign body’s location and the procedure’s technical complexity play a vital role in extracting the projectile. Removing the pellet, in this case, was deemed safe, given the proximity of the pellet to the injury site and ease of access. Optimising the operative planning is aided by radiological investigations. Many authors have recommended conventional angiography as a powerful modality allowing the detection of traumatic arterio-venous fistulas and pseudoaneurysms and its value in eliminating nontherapeutic neck explorations considering its high negative predictive value [16, 17]. Authors of a case report involving air rifle injury to the external carotid artery report facing unexpected intraoperative bleeding upon clearing a haematoma. They reflect on the utility of arteriography in identifying such potential risks to improve operative preparedness [18]. This opinion is challenged by some authors who believe angiography to be an invasive procedure with low diagnostic yield in patients with no hard signs of arterial injury [19]. Of note, Duplex USS was found to have a comparable diagnostic value to arteriography [16]. With the case at hand, CTA confirmed an arterial injury, raised the suspicion for a pseudoaneurysm, and localised the injury trajectory, enabling a clear operative plan. It also helped with identifying a thyroid injury. There is a lot of support for this diagnostic modality in penetrating neck injuries [20–22]. Therefore, we advocate using CTA for stable patients with PCI. A prospective study by Gonzalez *et al.* found that although CT studies did not add a diagnostic value to the physical examination in identifying arterial injuries, it proved helpful in diagnosing venous and oesophageal injuries [23].

The role of anticoagulation or antiplatelet therapy for PCI is not as clearly elucidated in the literature as it is for blunt carotid injuries [24]. There is also limited data on the superiority of antiplatelet versus anticoagulant agents in patients with no increased risk of bleeding [1]. Considering this data gap, our patient received three months of a single-agent antiplatelet medication based on a luminal injury repaired surgically to minimise the risk of embolic stroke. Prospective studies are needed to validate the efficacy of antiplatelet medication in PCI.

In conclusion, small-sized pellets from airguns involve a risk of embolization and vascular injury. Surgical neck exploration is the gold standard treatment for penetrating carotid injury. Operative planning before neck exploration is crucial and can be aided by CTA for haemodynamically stable patients.

## Data Availability

All the information provided in this manuscript is supported by clinical notes, formal reports, and images from the radiographic investigation modalities and clinical letters.
